# Comparison of Percutaneous Endoscopic Lumbar Discectomy and Open Lumbar Surgery for Adjacent Segment Degeneration and Recurrent Disc Herniation

**DOI:** 10.1155/2015/791943

**Published:** 2015-03-10

**Authors:** Huan-Chieh Chen, Chih-Hsun Lee, Li Wei, Tai-Ngar Lui, Tien-Jen Lin

**Affiliations:** ^1^Department of Neurosurgery, Wan Fang Hospital, No. 111, Section 3, Xinglong Road, Wenshan District, Taipei City 116, Taiwan; ^2^Graduate Institute of Injury Prevention and Control, Taipei Medical University, No. 250, Wuxing Street, Xinyi District, Taipei City 110, Taiwan

## Abstract

*Objective*. The goal of the present study was to examine the clinical results of percutaneous endoscopic lumbar discectomy (PELD) and open lumbar surgery for patients with adjacent segment degeneration (ASD) and recurrence of disc herniation. *Methods*. From December 2011 to November 2013, we collected forty-three patients who underwent repeated lumbar surgery. These patients, either received PELD (18 patients) or repeated open lumbar surgery (25 patients), due to ASD or recurrence of disc herniation at L3-4, L4-5, or L5-S1 level, were assigned to different groups according to the surgical approaches. Clinical data were assessed and compared. *Results*. Mean blood loss was significantly less in the PELD group as compared to the open lumbar surgery group (*P* < 0.0001). Hospital stay and mean operating time were shorter significantly in the PELD group as compared to the open lumbar surgery group (*P* < 0.0001). Immediate postoperative pain improvement in VAS was 3.5 in the PELD group and −0.56 in the open lumbar surgery group (*P* < 0.0001). *Conclusion*. For ASD and recurrent lumbar disc herniation, PELD had more advantages over open lumbar surgery in terms of reduced blood loss, shorter hospital stay, operating time, fewer complications, and less postoperative discomfort.

## 1. Introduction

Adjacent segment degeneration (ASD) is not a rare condition nowadays due to the fact that spinal surgery is more widely performed. This condition is defined as the recurrence of symptoms associated with the degeneration at the free segment above a fusion after symptom-free period. The incidence of symptomatic ASD is reported to be up to 30% [1]. In all ASD patients, herniated disc was the most common cause of repeated surgery.

The incidence of recurrent disc herniation has been reported to be around 5 to 18% in patients after open lumbar surgery [2–8]. Recurrent disc herniation is thought to be the major cause of surgical failure after open lumbar surgery, especially after microdiscectomy procedure. The generally accepted surgical management for recurrence of lumbar herniated disc is repeated open lumbar surgery; however, approach associated complications attributed to tissue scarring and adjacent segment degeneration caused by further damage to the vertebral motion segments should be considered [3, 9–11].

Furthermore, other than repeated open lumbar surgery, there may be other options to be considered. The endoscopic posterolateral transforaminal approach through intact tissue can avoid repeated damage to the posterior and paraspinal structures, making percutaneous endoscopic lumbar discectomy (PELD) a potential alternative to open lumbar surgery. PELD has already shown encouraging results for lumbar herniated disc [8, 12–20]; however, researches comparing clinical outcomes of repeated lumbar surgery by means of PELD with repeated open lumbar surgery are rare. In our study, we attempted to compare the surgical results of PELD and repeated open lumbar surgery for ASD and recurrent lumbar disc herniation.

## 2. Materials and Methods

### 2.1. Patient Demographics

**Figure 1:**
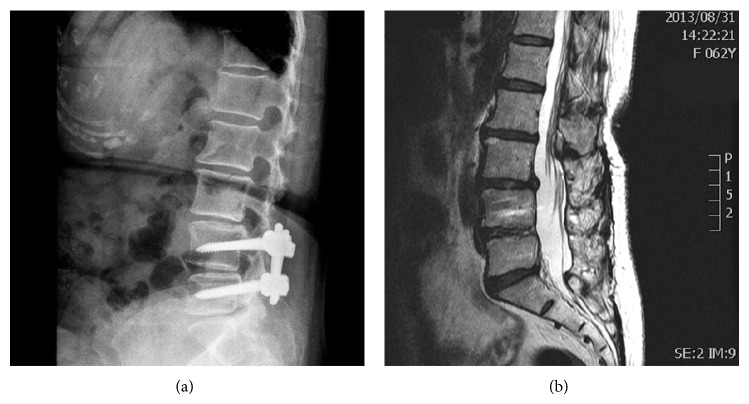
An illustrative patient who received open lumbar surgery at L4-5 level with adjacent segment degeneration at L3-4 level. (a) A lateral plain radiograph taken preoperatively showing previous internal fixation at L4-5 level. (b) Preoperative sagittal T2-weighted magnetic resonance imaging scan showing adjacent segment degeneration at L3-4 level.

From December 2011 to November 2013, we retrospectively collected forty-three patients who received repeated lumbar surgeries due to ASD or recurrent lumbar disc herniation. Inclusion criteria were (1) undergoing open lumbar surgery previously with or without fusion/fixation, (2) occurrence of recurrent or adjacent lumbar radicular pain subsequent to a painless period of at least four weeks, (3) herniated disc at the original level or the adjacent level, and (4) failed nonsurgical treatment for longer than six weeks [8, 21]. Exclusion criteria were patients who have serious neurological deficit and/or spinal instability. There were 26 men and 17 women, and the mean age of the patients was 55.9 years (range 25–80 years) (Figure 1).

Based on the surgical approaches, these patients were assigned to the following two groups. Group A included patients who received PELD, and Group B consisted of patients who underwent repeated open lumbar surgery for ASD and had recurrence of disc herniation. Six neurosurgeons were involved in our study, and the decision of surgical approach depended on each surgeon's expertise and preference. Among them, only one performed PELD for ASD and recurrence of disc herniation.

### 2.2. Surgical Procedures

In the open lumbar surgery group, the steps involved in the procedure were described to each patient before their operation. The surgeries were performed under general anesthesia in the prone position on a radiolucent operating table. A midline skin incision was made and paravertebral muscles were divided. Scar tissue was carefully removed to identify the previous laminotomy edges, and the surgeon was cautious not to tear the dura mater. Various surgical procedures were performed including removal of implants, microdiscectomy, laminotomy or laminectomy, and discectomy with/without fusion. Each wound was closed in layers after adequate decompression of the nerve roots.

**Figure 2:**
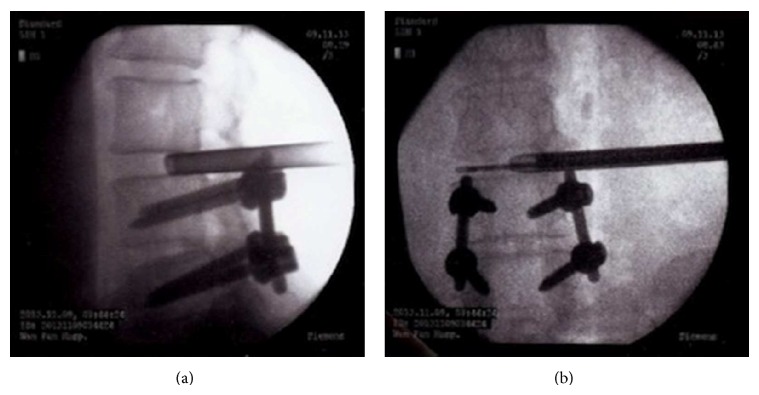
Fluoroscopic images taken intraoperatively. After insertion of endoscopic cannula (a), disc was removed using endoscopic forceps (b).

In the PELD group, patients were also informed of every step of the operation. The surgeries were carried out under local anesthesia in the prone position on a radiolucent operating table. During the entire procedure patients were able to communicate with the operator. The skin incision was made approximately 10 to 12 cm lateral from the midline. An 18-gauge spinal needle was inserted under fluoroscopic guidance after infiltrating the entry point with local anesthetics. The ideal target point of the spinal needle was the intersection of the posterior vertebral line on the lateral image and the medial pedicular line on the anteroposterior image. After the introduction of the spinal needle into the target disc space, the subsequent steps were as follows: we (1) inserted the guide wire through the spinal needle, (2) removed the spinal needle, (3) made a skin incision at the entry point, (4) inserted a tapered cannulated obturator along the guide wire, (5) inserted the obturator into the disc space with hammering after touching the annulus, and (6) inserted a oval-shaped, bevel-ended working cannula into the disc space along the obturator and then removed the obturator. The endoscope was inserted through the cannula and the disc was removed using endoscopic forceps working from the central to the lateral portion of the disc space on the anteroposterior image (Figure 2). After targeted fragmentectomy, the endoscope was retracted, the incision was sutured, and a sterile dressing was applied [14, 15].

### 2.3. Clinical Outcome Measures

Operative reports and medical charts were reviewed to obtain pre- and postoperative clinical data including operating time, total blood loss, and admission days. Pain was measured by the 10-point visual analogue scale (VAS) scoring (0 to 10) before and after surgery. Complications were categorized into major and minor using the classification scheme described by Carreon et al. [22]. Minor complications were considered not to have affected recovery significantly, whereas major complications were considered to have caused a negative effect on a patient's recovery.

Statistical methods were used to compare the patient demographic data and clinical outcomes of the two groups. The Student *t*-test was used on continuous parameters and the chi-square statistic test was used on categorical parameters. In the present study, *P* value <0.05 was considered to be statistically significant.

## 3. Results

Group A (PELD) included 18 patients and Group B (repeated open lumbar surgery) included 25 patients. There was no statistically significant difference in mean age at the time of surgery (57.4 in Group A and 54.9 in Group B, *P* = 0.597) and sex ratio (male : female, 12 : 6 in Group A and 14 : 11 in Group B, *P* = 0.697) (Table 1).

### 3.1. Clinical Outcomes

In Group A, the mean blood loss (minimal) was significantly less than that in Group B (303.2 mL, *P* < 0.0001). Mean operating time was shorter significantly in Group A (79.06 minutes) as compared with Group B (206.44 minutes, *P* < 0.0001). Mean hospital stay was shorter significantly in Group A (1.89 days) as compared with Group B (12.28 days, *P* < 0.0001) (Table 2).

No complications were encountered in the PELD group, but three minor complications occurred in the open surgery group in the perioperative period. Intraoperative incidental durotomy occurred in two patients and was managed successfully with primary suture. Wound infection occurred in one patient and required further debridement. After operation, immediate postoperative pain improvement in VAS was 3.5 in the PELD group as compared with −0.56 in the open lumbar surgery group (*P* < 0.0001) (Table 2). No ASD or recurrence occurred in the PELD group until now.

## 4. Discussion

PELD is a form of minimally invasive surgery and has many advantages over conventional open lumbar surgery. These advantages include performing the surgery under local anesthesia, preserved normal posterior and paraspinal structures, less postoperative pain, and early discharge [14]. For repeated lumbar surgery, not needing to go through the scar tissue could also be one significant advantage that is worth highlighting [13].

There are some studies which have discussed the use of PELD for patients with recurrence of disc herniation; however, there are still few relative discussions about the use of PELD for patients with ASD. A prospective cohort study performed by Hoogland et al. [13] evaluated a series of 262 patients who received PELD for recurrence of disc herniation. Both leg pain and back pain improved significantly, and the results of their surgeries were rated as excellent or good in 85.71% of the patients at 2-year follow-up. In another retrospective study, Xia et al.  [1] reported on 43 patients who underwent PELD for recurrent disc herniation. The mean VAS score decreased significantly, and based on the Macnab criteria 81.4% of the patients met excellent or good outcomes at a mean follow-up duration of 31 months. In our study, we compared the surgical results of PELD with those of repeated open lumbar surgery. PELD yielded clinical outcomes superior to repeated open lumbar surgery in terms of shortened operative time and admission days, decreased total blood loss, and lessened postoperative pain. Because normal paraspinal structures are preserved during PELD, it was thought to be superior to open lumbar surgery in terms of postoperative back pain. During repeated open lumbar surgery, dissection of paraspinal muscles and further destruction of posterior vertebral elements, such as medial facet joint and lamina, may heighten the risk of postoperative back pain [1, 10, 11]. Manchikanti et al. [23] reported that the prevalence of facet joint related chronic low back pain in postoperative group who received lumbar laminectomy was 32%. In our study, postoperative pain is significantly lower in the PELD group. As with PELD for primary lumbar herniated disc [17], PELD for recurrence of disc herniation as well had many advantages over repeated open lumbar surgery in that it could be carried out under local anesthesia, and the operating times as well as hospital stay were significantly shorter.

Regarding postoperative spinal stability, discectomy through transforaminal route has advantages over that through posterior route. As mentioned above, paravertebral and posterior spinal structures such as lamina, facet joint, ligaments and muscles could be preserved by means of transforaminal approach [1]. Osman et al. [24] reported one comparative study regarding postoperative stability of transforaminal and posterior approach using cadavers. After transforaminal decompression, there was minimal anatomic damage to the spine and there was no flexibility change. In contrast, there was significant increase in extension and axial rotation flexibility after the posterior decompression. Choi et al. [21] suggested that the atrophy and weakness of paraspinal muscles was one of the possible predisposing factors for further instability and dysfunction. In terms of paraspinal muscle atrophy, PELD was considered to have an advantage for ASD and recurrence of disc herniation since repeated open lumbar surgery had a higher chance of further damaging paraspinal muscles and innervating dorsal rami. However, Lee et al. [8] suggested that the influences of second surgeries on paraspinal muscles were not significant because denervation atrophy of paraspinal muscles had already significantly progressed after the first open lumbar surgery.

Approach related complication is a major concern for repeated lumbar surgery. Epidural adhesion and scar tissue often make repeated open lumbar surgery difficult and thus increase the risk of nerve root injury or intraoperative incidental durotomy [3, 9–11, 20]. The incidence of intraoperative incidental durotomy was reported to be up to 20% in patients who received repeated open lumbar surgery [8, 25, 26]. It was suggested that intraoperative incidental durotomy during lumbar surgery could result in poorer clinical outcomes and long-term neurological sequelae [27]. In contrast, considering repeated lumbar surgery by means of PELD, some studies reported encouraging outcomes. Xia et al. [1] Hoogland et al. [13] and Lee et al. [8] reported that there was no case of intraoperative incidental durotomy or cerebrospinal fluid leakage after surgery in 43, 262, and 23 consecutive patients, respectively. In our study, there was also no intraoperative incidental durotomy in PELD group, whereas intraoperative incidental durotomy occurred in two patients (8%) in the group of repeated open lumbar surgery.

Injury of nerve root during repeated lumbar surgery may result in long-term neurological sequelae and poorer clinical outcome. Choi et al. [21] reported that one (2.9%) of 35 patients developed permanent foot drop after repeated open lumbar surgery. Lee et al. [8] also reported that there was one (3.4%) patient who developed persistent voiding disturbance along with dysesthesia after repeated open lumbar surgery in 28 consecutive patients. Considering repeated lumbar surgery by means of PELD, Hoogland et al. [13] reported three patients (1.1%) with nerve root irritation and Xia et al. [1] reported one patient (2.3%) with transient dysesthesia, but no patient suffered permanent injury of nerve root in either series. Lee et al. [8] reported that there was no case of nerve root injury after PELD in 23 consecutive patients. In our study, there was no nerve root injury observed in both PELD and repeated open lumbar surgery group.

Although our study showed promising outcomes, the relatively small number of patients and the retrospective study design of this study should be taken into consideration when interpreting our results. There is still a need for further randomized controlled trials.

## 5. Conclusion

For patients with ASD and recurrent disc herniation, both PELD and repeated open lumbar surgery showed favorable clinical outcomes in the present study. Furthermore, PELD had several advantages over repeated open lumbar surgery in terms of what follows: (1) the procedure being possibly performed under local anesthesia, (2) significantly shortened operating time, (3) significantly less mean blood loss, (4) no complications related to surgical approach, such as dural tear and/or nerve root injury, (5) less postoperative discomfort, and (6) significantly decreased number of admission days.

Overall, the PELD group achieved the goal of early mobilization and a shorter postoperative period to normal daily activity. In the long run, the increased use of this type of procedure may reduce the economic burden of this disease for patients, their families, and society in general.

## Figures and Tables

**Table 1:** Summary of patient demographic data for the two groups.

	PELD	Open lumbar surgery	*P* value
Age (y/o)	57.4 ± 12.4	54.9 ± 16.6	0.597
Gender (M : F)	12 : 6	14 : 11	0.697

**Table 2:** Summary of clinical outcomes who underwent PELD (Group A) and open lumbar surgery (Group B).

	Group A	Group B	*P* value
Operating time (minutes)	79.06 ± 31.05	206.44 ± 74.19	<0.0001
Total blood loss (mL)	Minimal	303.2 ± 266.62	<0.0001
Admission days (days)	1.89 ± 0.74	12.28 ± 7.58	<0.0001
Postoperative pain improvement (VAS)	3.5 ± 2.41	−0.56 ± 3.01	<0.0001
